# The Effects of Uygur Herb *Hyssopus officinalis* L. on the Process of Airway Remodeling in Asthmatic Mice

**DOI:** 10.1155/2014/710870

**Published:** 2014-10-15

**Authors:** Xiaojuan Ma, Xiumin Ma, Zhixing Ma, Zhan Sun, Wenyan Yu, Jing Wang, Fengsen Li, Jianbing Ding

**Affiliations:** ^1^Xinjiang National Clinical Research Base of Traditional Chinese Medicine, Xinjiang Medical University, Urumqi, Xinjiang 830000, China; ^2^College of Basic Medicine, Xinjiang Medical University, Urumqi, Xinjiang 830011, China; ^3^Department of Clinical Laboratory, First Affiliated Hospital of Xinjiang Medical University, Urumqi, Xinjiang 830011, China; ^4^Urumqi General Hospital of Lanzhou Military Area Command, Urumqi 830000, China

## Abstract

It has been proved that Uygur herb *Hyssopus offcinalis* L. could affect the levels of some cytokines (such as IL-4, IL-6, IL-17, and IFN-*γ*) in asthmatic mice. By detection of the expressions of MMP-9 and TIMP-1 and the morphological changes, the aim of this research is to reveal the mechanism of Uygur herb *Hyssopus offcinalis* L. in the process of airway remodeling. It was observed that the expressions of MMP-9 and TIMP-1 increased, but the ratio of MMP-9/TIMP-1 decreased in airway remodeling group. However, the expression of both MMP-9 and TIMP-1 decreased after being treated with dexamethasone and *Hyssopus offcinalis* L., accompanied by the relieved pathological changes, including collagen deposition, mucus secretion, and smooth muscle proliferation. It is suggested that Uygur herb *Hyssopus offcinalis* L. could inhibit airway remodeling by correcting imbalance of MMP-9/TIMP-1 ratio.

## 1. Introduction

Asthma is a common clinical disease [1, 2]. The epidemiological survey has shown that its incidence, mortality, and morbidity are increasing all over the world. In China, the incidence of asthma has doubled over the past decade. Present treatment of asthma in clinic is anti-inflammatory-based combination therapy. However, some patients, especially hormone resistant ones, are not sensitive to this method.* Hyssopus officinalis *L., a kind of Uygur medicine Hyssop, is a perennial herb of Labiatae that could relieve cough and asthma. Previous studies have shown that* Hyssopus officinalis *L. may play anti-inflammatory role through regulating the secretions of IL-4, IL-6, IL-17, and interferon-*γ* (IFN-*γ*) and correcting the imbalance between Th1/Th2 cells [3–6].

The pathogenesis of asthma has been not clear till now, but the main pathological feature is airway remodeling. It was indicated that the histological hallmarks of chronic inflammation and remodeling are as follows: (1) infiltration by macrophages and lymphocytes; (2) proliferation of fibroblasts that may take the form of myofibroblasts; (3) angiogenesis; (4) increased connective tissue (fibrosis); and (5) tissue destruction. It is clear that changes in extracellular matrix (ECM), smooth muscle, and mucous glands have the capacity to influence airway function and reactivity in asthma patients [7]. The main reason of fibrosis and airflow obstruction may be the deposition of ECM in the wall of airways. Matrix metalloproteinase (MMP) and tissue inhibitors of metallo proteinases (TIMPs) are crucial enzymes in the regulation of ECM metabolism, especially MMP-9 and TIMP-1. Han et al. indicated that MMP-9 was expressed by bronchial epithelium and may be an important factor for airway eosinophil infiltration in asthma subjects [8]. Zhu et al. further demonstrated that the expression of MMP-9 was increased in acute asthma model, but the expression of TIMP-1 rose in the remodeling model of airways [9].

The function of* Hyssopus officinalis *L. on the pathogenesis of airway remodeling, however, has not been reported. We hypothesise that* Hyssopus officinalis *L. may influence the airway remodeling pathogenesis via regulation of MMP-9/TIMP-1 ratio.

## 2. Materials and Methods

### 2.1. Chemicals and Reagents

Synthesized M-MLV first-strand reverse transcription system kit was purchased from Invitrogen Life Technologies (USA). The primers have been designed and synthesized by the Jiancheng Bioengineering Institute of Nanjing (Nanjing, China). MMP-9 antibody, TIMP-1 antibody, and pierce goat anti-rabbit IgG were obtained from Boster Biological Engineering Co., Ltd. (Wuhan, China). Masson Staining Kit and PAS Staining Kit were provided by Senbeijia Biotechnology Co. (Nanjing, China).

### 2.2. Preparation of Extract

200 g crude herbs were dissolved in 6000 mL water. The solvents were extracted via rotary evaporation under reduced pressure. The aqueous extract was further frozen and dried and then stored at 4°C. Aqueous extract was prepared for use after being diluted with saline water.

### 2.3. Acute Toxicology Test

In our previous study, we had done the acute toxicology test and refer to the body surface area calculation method in Pharmacology Experiment Instruction. Healthy mice (*n* = 20) were orally fed with increasing doses (0.04 g/10 g to 1.6 g/10 g body weight) of extracts for 14 days. The doses up to 1.6 g/10 g body weight did not produce any toxicity and mortality. The maximum-tolerated dose for mice is 1.60 g/10 g. At the same time, we also found that the effect of high-dose group was better than low-dose group. So 0.04 g/10 g was selected for the study [3–6].

### 2.4. Animals and Modeling

Thirty-two female BALB/c mice (ordered from Animal Experiment Center, Xinjiang Medical University, Xinjiang) were housed in microisolator cages with water and food supply. The laboratory temperature was 24 ± 1°C, and the relative humidity was 40–80%. All experimental protocols were approved by the regional Animal Ethics Committee.

Thirty-two female BALB/c mice (6–8 weeks old) were randomly divided into four groups: control, airway remodeling, dexamethasone, and* Hyssopus officinalis *L. groups. Except control group, the other three groups received intraperitoneal injection of sensitizing agent 0.2 mL (containing OVA 100 *μ*g and aluminum hydroxide gel 1 mg) on day 1 and day 15, respectively, and then breathed in 1% OVA for 30 min that started from day 22, 3 times per week for 8 weeks during which the animals were daily administered with the drugs via intragastric tube (dexamethasone 0.005 mg/10 g,* Hyssopus officinalis *L. 0.04 g/10 g). Control group was treated with PBS instead of OVA and with normal saline via intragastric tube [10].

Animals were euthanized after the last challenge and specimens were collected.

### 2.5. The Expressions of MMP-9 and TIMP-1 mRNA

Total RNA were extracted from lung tissue. The cDNAs were synthesized. The primers were designed as follows: 5′-caaagacctgaaaacctccaac-3′ (forward) and 5′-gactgcttctctcccatcatct-3′ (reverse) for MMP-9; 5′-accgcagtgaagagtttctca-3′ (forward) and 5′-atccgtccacaaacagtgagt-3′ (reverse) for TIMP-1; 5′-tgttaccaactgggacgaca-3′ (forward) and 5′-ggggtgttgaaggtctcaaa-3′ (reverse) for *β*-actin. The real-time PCR was carried out in a 20 *μ*L final volume container and performed by an initial denaturation at 95°C for 2 min, followed by 40 cycles of annealing temperatures of 55°C (MMP-9, TIMP-1) and 50°C (*β*-actin), respectively. The electrophoretic bands of amplification product were scanned by using the gel imaging analysis system. The expression of MMP-9 mRNA is the ratio of MMP-9 optical density and beta-actin 1 optical density, and the expression of TIMP-1 mRNA is the ratio of TIMP-1 optical density and beta-actin 2 optical density.

### 2.6. The Expressions of MMP-9 and TIMP-1 in the Level of Protein

After euthanasia, the lungs were removed and grinded. Extracting the tissue protein was followed. Western-blot assays were performed as previously described [11, 12].

### 2.7. The Pathological Changes

The left lung was removed and put into 10% formaldehyde solution followed by dehydration, paraffin embedding, sectioning, and hematoxylin-eosin staining sequentially. The changes of pathology were observed.

### 2.8. The Measure of Morphology

Lung tissue sections stained by HE were observed to study morphological alternation in terms of total area of the bronchial wall (WAt), airway smooth muscle area (WAm), and inner way area (WAi). The ratio of those indexes and bronchial basement membrane perimeter (Pbm) was compared in different groups.

### 2.9. Deposition of Collagen in the Lung Tissue

Masson's trichrome was used to determine collagen deposition in the lungs [13] and to identify Masson staining area around the airway and the average length of basement membrane was identified. A minimum of 10 fields throughout the upper and lower right lung tissue were randomly examined for the morphometric analyses by Nikon Eclipse Ci (Japan).

### 2.10. Secretion of Mucus in Lung

PAS staining was used to determine the secretion of mucus. The goblet cells were stained because of glycoprotein. The positive cell was in purple. A minimum of 10 fields throughout the upper and lower right lung tissue were randomly examined. The ratio of positive cell to epithelial cell was calculated and evaluated as follows: 0 score <5%; l score 5–25%; 2 score 25–50%; 3 score 50–75%; 4 score >75%.

### 2.11. Statistical Analysis

SPSS17.0 statistical software was used to analyse. The data was presented as the means ± SEM. Comparison between groups was made with ANOVA followed by Dunnett's test. *P* values of 0.05 or less were considered statistically significant.

## 3. Results 

### 3.1. The Changes of Behavior

The mice presented with the following symptoms after challenge by OVA, including anxiety, frequent nose scratching, cough, nodding breathing, shortness of breath, retardation, piloerection, and cyanosis. The activities were decreased after continuous challenge. But these symptoms were relieved in dexamethasone group and* Hyssopus officinalis *L. group (Table 1).

### 3.2. The Expressions of MMP-9 and TIMP-1 mRNA

**Figure 1:**
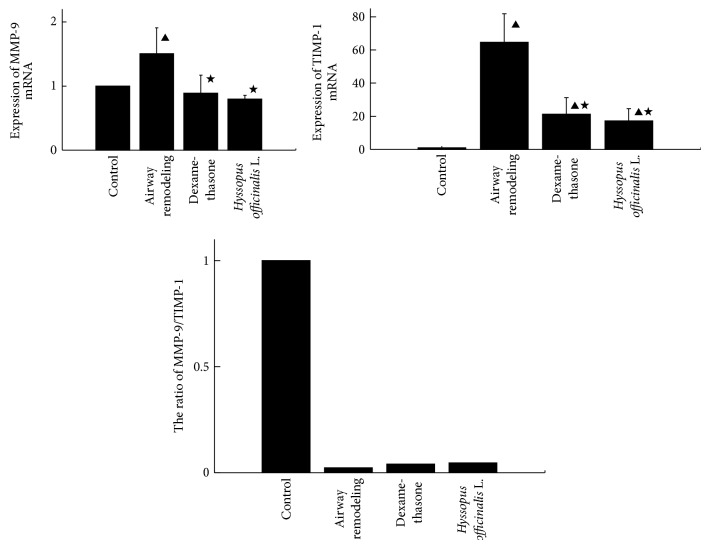
Expressions of MMP-9, TIMP-1, and MMP-9/TIMP-1 mRNA in different groups. ▲*P* < 0.05 versus control group; ★*P* < 0.05 versus airway remodeling group.

As shown in Figure 1, significant increases of MMP-9 and TIMP-1 mRNA expressions were detected in airway remodeling group compared with control group (*P* < 0.05); but the expressions of both MMP-9 and TIMP-1 mRNA decreased obviously in dexamethasone group and* Hyssopus officinalis *L. group (*P* < 0.05).

Moreover, the ratios of MMP-9/TIMP-1 mRNA expressions were decreased in airway remodeling group compared with control group, while increased in dexamethasone group and* Hyssopus officinalis *L. group compared with airway remodeling group.

### 3.3. The Expressions of MMP-9 and TIMP-1 in the Level of Protein

**Figure 2:**
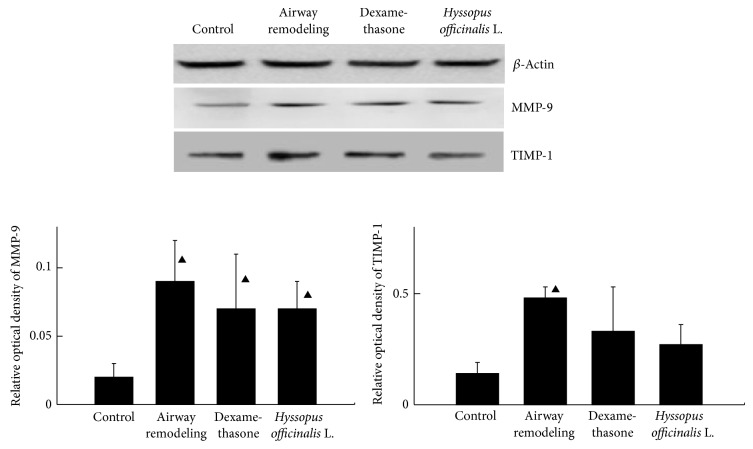
Expressions of MMP-9 and TIMP-1 proteins in the lung. ▲*P* < 0.05 versus control group.

The extracted lung tissue proteins were assayed by Western-blot analysis. As shown in Figure 2, the contents of MMP-9 and TIMP-1 proteins were increased in airway remodeling group compared with control group (*P* < 0.05); but the contents were decreased in dexamethasone and* Hyssopus officinalis *L. groups.

### 3.4. The Pathological Changes

**Figure 3:**
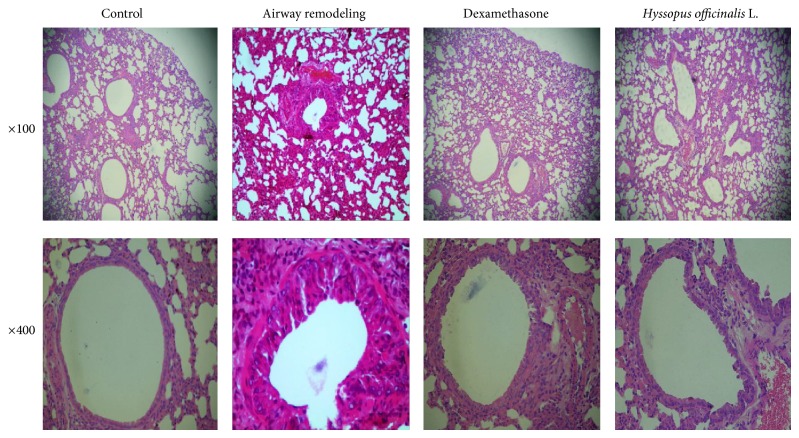
Changes of lung tissue after HE staining.

As shown in Figure 3, airway remodeling group presented with changes, including narrowed airway, mucus plug formation, thickened airway smooth muscle, shed epithelial cells, increased number of goblet cells, and obvious inflammatory cell infiltration into submucosa. However, all the above changes were relieved in dexamethasone group and* Hyssopus officinalis *L. group.

### 3.5. The Measure of Morphology

**Figure 4:**
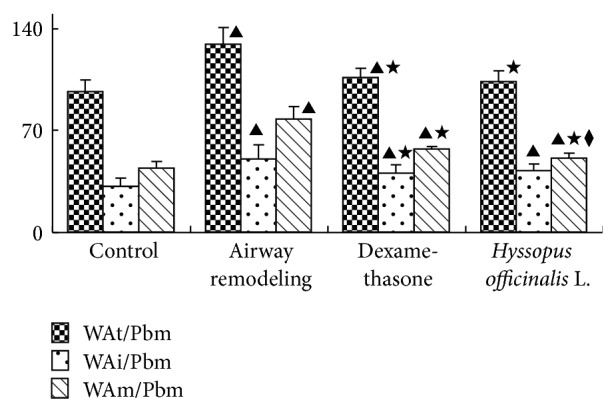
The change of morphology. ▲*P* < 0.05 versus control group; ★*P* < 0.05 versus airway remodeling group.

It will reflect the thickness of epithelia and airway diameter. It can be seen in Figure 4 that the change of WAt/Pbm, WAi/Pbm, and WAm/Pbm has the same tendency. Those indexes are significantly higher in the other three groups compared to a control group (*P* < 0.05); WAt/Pbm, WAi/Pbm, and WAm/Pbm all reduced in dexamethasone group and* Hyssopus officinalis *L. group compared with airway remodeling group (*P* < 0.05).

### 3.6. Deposition of Collagen in Lung Tissue

**Figure 5:**
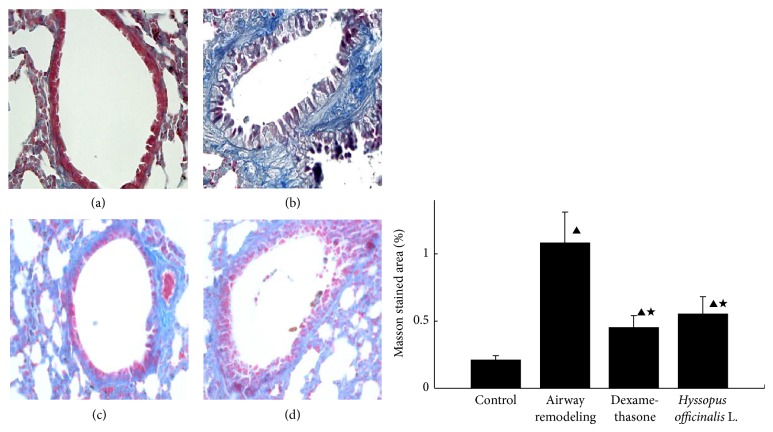
The comparison of collagen deposition in different groups ((a) control group; (b) airway remodeling group; (c) dexamethasone group; (d)* Hyssopus officinalis *L. group). ▲*P* < 0.05 versus control group; ★*P* < 0.05 versus airway remodeling group.

Compared with control group, dense collagen deposition/fibrosis was seen throughout the lung interstitium surrounding the airways and blood vessels in airway remodeling group (Figure 5), and the ratio of staining area/length of basement membrane was significantly increased (*P* < 0.05); but treatment with dexamethasone and* Hyssopus officinalis *L. markedly reduced collagen deposition in the lung interstitium of asthmatic mice and the ratio staining area/length of basement membrane (*P* < 0.05).

### 3.7. Secretion of Mucus in Lung

**Figure 6:**
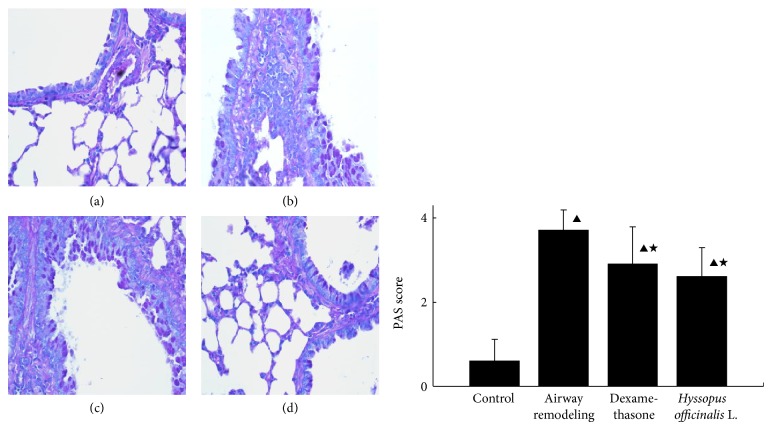
The comparison of mucus secretion in different groups ((a) control group; (b) airway remodeling group; (c) dexamethasone group; (d)* Hyssopus officinalis *L. group). ▲*P* < 0.05 versus control group; ★*P* < 0.05 versus airway remodeling group.

It can be seen in Figure 6 that there were less positive epithelial cells in control group via PAS staining. The percentage of positive cells to total epithelial cells was the highest in airway remodeling group, while declined in dexamethasone and* Hyssopus officinalis* L. groups (*P* < 0.05).

## 4. Discussion

Airway remodeling can be considered a collective term and encompasses the alterations in structural cells as well as tissues in the asthmatic opposed to the normal airway [14]. It will lead to irreversible airflow obstruction and damage pulmonary function. Many reports have highlighted that the consequences of the wall thickening, subepithelial fibrosis, mucus metaplasia, myofibroblast and myocyte hyperplasia, and epithelial hypertrophy are the characteristic in response of remodeling. The deposition of excessive ECM in the airway wall can result in airway wall fibrosis [15]. MMPs, especially MMP-9, are extracellular proteases which will degrade the ECM during remodeling of the tissues. Song's team showed that the activity of MMP-9 increased in asthma group, and 1, 25-(OH)_2_-D_3_ could decrease its expression [16]. Studies with MMP-9-deficient mice challenged by allergen have demonstrated that they had modest reductions in peribronchial fibrosis but no reduction in mucus expression, smooth muscle thickness, or airway responsiveness [17].

Our results showed that the expression of MMP-9 mRNA in airway remodeling group was higher than that in control group; moreover, the expression of MMP-9 in the level of protein also increased. It is known that a variety of cells (including epithelial cells, endothelial cells, eosinophils, etc.) could release MMP-9 because of allergens. MMP-9 may further activate the matrix related growth factors, such as TNF-*α*, epidermal growth factor, platelet-derived growth factor, and insulin-like growth factor. TNF-*α* will promote not only the migration of inflammatory cells to the affected part but also the proliferation of airway smooth muscle. Through observing and measuring the lung tissues, it can be seen that the thickness of epithelia in airway remodeling group was increased significantly, while the airway diameter was notably decreased in airway remodeling group (*P* < 0.05). Moreover, since MMP-9 might combine with CD44 to produce TGF-*β* which could increase the collagen synthesis of fibroblasts, excessive collagen deposits in airway remodeling group were also observed in this study by Masson staining. Besides, increased mucus secretion that formed mucus plug in airway remodeling group suggests a possible association between MMP-9 and mucus secretion.

A pivotal extracellular control of MMP catalytic activity is accomplished by members of a specific family of inhibitors named TIMPs [18]. Some researchers thought that MMP-9/TIMP-1 ratio may play a key role in the destruction of bronchial tissues and its repair [19, 20].

Our results showed that the expression of TIMP-1 mRNA and its proteins was more obvious than that of MMP-9 in airway remodeling group, leading to a prominent decrease in the ratio of MMP-9/TIMP-1. The excessive expression of TIMP-1 will inhibit the activity of MMP-9, resulting in excessive ECM deposits in the airway because of an imbalance between the degradation of bronchial matrix protein and the restoration of tissue. In addition, by regulating matrix related growth factors, the decline of MMP-9/TIMP-1 ratio could promote the airway smooth muscle proliferation. Thus, the ratio of MMP-9/TIMP-1 could be used as a parameter reflecting the balance between the destruction and the restoration of airway tissue [21, 22].

Some traditional Chinese medicinal herbs have demonstrated efficacy in both mouse models and patients of allergic asthma, such as ASHMI [23]. The researcher found that mesenchymal stem cells (MSCs) could improve the liver fibrosis through modulating the balance of MMP and TIMP to decrease the collagen deposition. The traditional Chinese medicine also could regulate the development of MSCs [24].

*Hyssopus officinalis *L., a kind of Uygur medicine, is used to cure asthma, cough, fever, and rheumatism [25]. A large number of studies focusing on this drug have been done by our research group. It was shown that* Hyssopus officinalis *L. could influence the expression of some cytokines in the asthmatic mice [3–6].

In this study, it was revealed that the expressions of both MMP-9 and TIMP-1 were decreased in* Hyssopus officinalis *L. group, and changes of morphology were less than airway remodeling group. It was suggested that* Hyssopus officinalis *L. could relieve the ECM deposition by adjusting the ratio of MMP-9/TIMP-1 and inhibiting the smooth muscle thickening, goblet cells hyperplasia, and fibrosis.

In conclusion, whether* Hyssopus officinalis *L. will regulate MMP-9/TIMP-1 ratio via affecting the expressions of some cytokines (such as IL-1, IL-17, and TNF-*α*) or not still needs to be further illuminated. By revealing the effect of* Hyssopus officinalis *L. on airway remodeling, our study may provide a theoretical foundation for treating airway remodeling with Chinese medicine.

## Figures and Tables

**Table 1:** The change of behavior in different groups.

	Anxiety	Frequent nose scratching	Cough	Nodding breathing	Shortness of breath	Retardation	Piloerection	Cyanosis
Control group	− −	− −	− −	− −	− −	− −	− −	− −
Airway remodeling	++	++	++	++	++	++	++	++
Dexamethasone	+−	+−	+−	+−	+−	+−	+−	+−
*Hyssopus officinalis *L.	+−	+−	+−	+−	+−	+−	+−	+−
